# Supplementary material for: A Likelihood Approach for Real-Time Calibration of Stochastic Compartmental Epidemic Models
Source: PLoS Comput Biol. 2017 Jan 17;13(1):e1005257. doi: 10.1371/journal.pcbi.1005257 (PMC5240920; doi:10.1371/journal.pcbi.1005257)
Supplement: S1 File — (TAR.GZ) [file pcbi.1005257.s014.tar.gz › HSPH_Online-SI-Revision/PF08/n10-severe/n10-severe_PF2_summary2.pdf]

peak −8	3w spec	3w cum	5w spec	5w cum	end	3w spec LOG	3w cum LOG	5w spec LOG	5w cum LOG	end LOG
mean	148.6	157.	58.6	77.5	22.	0.3	0.3	0.3	0.2	0.1
median	52.4	62.2	48.	46.7	23.6	0.2	0.2	0.3	0.2	0.1
quantile .1	20.8	21.8	26.7	19.5	8.4	0.1	0.1	0.1	0.1	0.
quantile .9	311.4	429.9	100.1	164.1	35.1	0.6	0.6	0.4	0.1	0.2
47	37	41	48	46						

peak −4	3w spec	3w cum	5w spec	5w cum	end	3w spec LOG	3w cum LOG	5w spec LOG	5w cum LOG	end LOG
mean	43.2	39.8	38.3	40.3	21.6	0.3	0.2	0.2	0.3	0.1
median	42.5	38.3	38.2	40.2	21.7	0.3	0.2	0.2	0.2	0.1
quantile .1	22.1	24.1	21.9	21.8	6.9	0.1	0.1	0.1	0.1	0.
quantile .9	60.8	54.5	56.9	57.3	35.4	0.4	0.4	0.4	0.1	0.1
11	12	16	12	36						

peak	3w spec	3w cum	5w spec	5w cum	end	3w spec LOG	3w cum LOG	5w spec LOG	5w cum LOG	end LOG
mean	39.9	22.9	113.1	32.7	77.9	0.1	0.1	0.3	0.1	0.2
median	29.3	22.4	78.5	31.	75.6	0.1	0.1	0.2	0.1	0.2
quantile .1	7.4	7.7	17.	6.6	29.6	0.	0.	0.1	0.	0.1
quantile .9	85.4	40.2	201.9	63.2	125.3	0.3	0.2	0.5	0.	0.4
14	11	2	8	1						

peak +4	3w spec	3w cum	5w spec	5w cum	end	3w spec LOG	3w cum LOG	5w spec LOG	5w cum LOG	end LOG
mean	250.6	142.6	799.	182.	279.2	0.4	0.3	0.6	0.4	0.5
median	151.9	98.6	309.1	125.4	205.3	0.4	0.3	0.6	0.3	0.5
quantile .1	48.8	38.4	90.8	51.3	87.4	0.2	0.1	0.3	0.2	0.3
quantile .9	438.5	255.8	1387.1	309.9	455.1	0.7	0.5	1.2	0.2	0.7
1	0	0	1	0						

peak +8	3w spec	3w cum	5w spec	5w cum	end	3w spec LOG	3w cum LOG	5w spec LOG	5w cum LOG	end LOG
mean	12 694.6	1436.7	13 055.1	1869.8	2553.4	1.	0.7	1.1	0.8	0.9
median	462.3	307.6	582.3	339.4	533.	0.7	0.6	0.8	0.6	0.8
quantile .1	122.5	92.9	138.7	107.6	155.4	0.3	0.3	0.4	0.3	0.4
quantile .9	5043.5	1814.7	78 690.	2039.	2687.6	1.7	1.3	2.9	0.3	1.4
0	0	0	0	0	{}	{}	{}	{}	{}	{}
